# Healthcare Providers’ Perspectives on Autistic Adolescents Transitioning to Independent Driving

**DOI:** 10.1007/s10803-024-06626-6

**Published:** 2024-11-15

**Authors:** Catherine C. McDonald, Christina Labows, Rachel K. Myers, Emma Sartin, Benjamin E. Yerys, Meghan E. Carey, Cynthia J. Mollen, Allison E. Curry

**Affiliations:** 1 School of Nursing, University of Pennsylvania, 418 Curie Blvd. Claire Fagin Hall, Room 414, Philadelphia, PA USA; 2 Center for Injury Research and Prevention, Children’s Hospital of Philadelphia, Philadelphia, PA USA; 3 Department of Pediatrics, Perelman School of Medicine, University of Pennsylvania, Philadelphia, PA USA; 4 Center for Autism Research, Children’s Hospital of Philadelphia, Philadelphia, PA USA; 5 Department of Psychiatry, Perelman School of Medicine, University of Pennsylvania, Philadelphia, PA USA; 6 Division of Emergency Medicine, Department of Pediatrics, Perelman School of Medicine, University of Pennsylvania, Philadelphia, PA USA; 7 PolicyLab, Children’s Hospital of Philadelphia, Philadelphia, PA USA; 8 School of Public Health, University of Alabama at Birmingham, Birmingham, AL USA

**Keywords:** Autism spectrum disorder, Adolescent health, Transportation, Licensure, Parents, Healthcare providers

## Abstract

Licensure is an option for some autistic adolescents and families that increases mobility by enabling independent travel to employment, school, and social activities. The objective of this study was to identify current strategies used by healthcare providers (HCPs) in their guidance to autistic adolescents and families on the transition to independent driving. Semi-structured interviews were conducted with 15 HCPs. The team’s previous research, literature review and expert feedback informed the development of the interview guide. A content analysis approach was used in the coding of transcripts, nine of which were double coded. Study team members reviewed coded transcripts, provided and discussed narrative summaries, and identified themes. Interviews were conducted with physicians, social workers, psychologists, therapist and a nurse practitioner. HCP identified their perceptions of autistic adolescents’ strengths and weaknesses to be addressed in their individualized approaches. They described using clinical interactions as time to address licensure and driving, but also referred to specialists as needed. HCPs described using existing resources, but also provided a wish list of future resources. HCPs use an individualized approach for guidance in the transition to independent driving, considering the unique needs of autistic adolescent patients and families. These HCPs identified a clear need for tailored resources and guidance they can use in support of independent driving when appropriate for their patients and families.

## Introduction

The transition to adulthood is a particularly challenging time for autistic adolescents. The 2021 National Autism Indicators Report states that only about one third of autistic adults who live with their families had paid employment, 17% attended some type of school, and nearly half of family respondents said their autistic adult stays at home throughout the day (Roux et al., 2021). An underlying reason for these poor quality of life outcomes is limited community mobility (Wilson et al., 2021). Parents view transportation as critical to connecting their autistic adult children to interventions and activities (Lubin & Feeley, 2016).

Licensure is an option for some that increases mobility by enabling independent travel to employment, school, and social activities. A 2017 study indicated that one in three autistic adolescents acquired a driver’s license by age 21; nearly 90% of those who acquired a permit went on to get licensed within 2 years (Curry et al., 2017). Further, licensed autistic adolescents and young adults are not involved in crashes any more frequently than their non-autistic peers (Curry et al., 2021). Parents believe their autistic adolescents are interested in driving and autistic adults who receive a license report more positive quality of life outcomes (Huang et al., 2012; Wilson et al., 2021). There is a need to better understand how licensing decisions are made, as well as the perception, attitudes, and experience of those who directly support these adolescents—namely parents, driving educators, and healthcare providers.

Healthcare providers (HCPs) working with autistic adolescents occupy an important space in the transition to adulthood. This can include transitioning to adult health care services and addressing young adulthood developmental milestones. Prior research shows that families trust HCPs to provide information, particularly on adolescent driving (Ford et al., 2016). However, a recent survey of HCPs found only about 20% discuss transportation with autistic patients, compared with 50% with their non-autistic peers (Sartin et al., 2021). Further, only 8% of providers felt prepared to provide guidance and assess driving readiness among autistic patients, highlighting a critical gap in available resources.

As part of a larger research program to examine the transition to independent driving among autistic adolescents from the perspectives of multiple stakeholders, we conducted a qualitative study with HCPs who delivered care to autistic patients and families. In a prior manuscript, (Myers et al., In Press) reports on HCPs’ perspectives regarding their role in supporting autistic adolescents and caregivers in the decision to pursue driving and identifying specific barriers HCPs encounter in providing this support. This manuscript builds on those prior results to identify the strategies currently used by HCPs to provide guidance to autistic adolescents and families and their recommendations for resource development to aid in this transition. Results will directly inform the development of future resources and guidance that can be used and delivered by HCPs in support of independent driving.

## Methods

### Study Design & ParticipantS

Our team conducted semi-structured interviews to explore how HCPs provide guidance to their autistic patients on the topics of driving and transportation. We recruited participants through an interest question at the conclusion of a previous study’s quantitative survey (Sartin et al., 2021). The population contacted included HCPs at the Children’s Hospital of Philadelphia (CHOP), the Philadelphia Autism Centers of Excellence, and providers recommended for participation by other participants via snowball sampling. This study was determined to be exempt by the CHOP Institutional Review Board.

We developed the interview guide by integrating information from the authors’ previous research with other stakeholders such as specialized driving instructors (Ford et al., 2016; Huang et al., 2012), literature review, and feedback from two external experts in the field. The interview guide was reviewed by professionals external to the team with expertise in driving, autism, and the transition to adulthood, to provide feedback on clarity and appropriateness of questions. The interview guide covered topics to elicit providers’ experiences providing guidance to parents and autistic adolescents regarding driving and licensure, challenges and barriers, strengths and concerns around autistic adolescent drivers, discussion of non-driving related transportation activities, and resources. Appendix A in Supplementary Materials includes the HCP interview guide with main questions and example probes.

The interviewer (CL) trained with the team’s qualitative experts (CJM and RKM) and conducted all fifteen interviews over the phone with supplementary notes. Interviews were audio-recorded and transcribed. The team’s qualitative experts reviewed the first four transcripts, provided feedback, and suggested minor changes to the interview guide to clarify questions. The team met biweekly to discuss updates from the interviewer about the patterns of the interview content and identify saturation. The team determined that the interviews had reached saturation when consistent patterns were identified in participant responses and no new information arose from interviews. Interviews ranged from 21 to 38 min, with an average of 30.2 min. Participants received $20 for participation.

### Data Analysis

The team used a content analysis approach to develop a codebook based on previous literature and interview guide. The codebook included the name of the code, a definition describing when to apply the code, and when not to use the code. As the team proceeded with coding, key examples were added to the codebook for each code to assist in accurate and consistent coding. The codebook also included coding rules established by the team, such as coding in full sentences, and including enough context in coded text to inform a naïve reader.

Interviews were de-identified and imported into NVivo (Version 11) (QSR International, 2016). Two team members (CL and LL) reviewed four transcripts independently which were assessed for inter-rater reliability (using Cohen’s K). The two coders met to address discrepancies for codes that did not meet an inter-rater reliability of K > 0.8 and met with neutral third parties (MC and RKM) to come to consensus on any discrepancies that could not be resolved between the two coders. Following adjustments and finalization of the codebook, the two coders recoded the original four transcripts, reassessed inter-rater reliability, and once the codebook was able to be applied independently and consistently, the coders continued with additional transcripts. The remaining transcripts were coded by one of the two trained team members, with nine double coded.

Five team members with various areas of expertise reviewed coded text, provided narrative summaries, and discussed these summaries. The themes presented herein arose from the discussion of the narrative summaries.

## Results

Interviews were conducted with 15 HCPs. Table 1 details the participant demographics. The double coded transcripts had a mean K = 0.87 with a range of 0.82–0.97. Three themes related to strategies used by HCPs emerged from the data: (1) Addressing the Individual Needs of Patients and Families ; (2) In-the-Moment Guidance and Referral Out; (3) Resources Present and Future.

**Table 1:** Participant demographics (*N* = 15)

Characteristics	*N* (%)
Female	13 (86.7%)
Race	
Black or African American	3 (20.0%)
White	12 (80.0%)
Hispanic or Latino	1 (6.7%)
Years in Practice	
0–4 years	5 (33.3%)
5–10 years	4 (26.7%)
11–15 years	0 (0%)
16–20 years	1 (6.7%)
> 20 years	5 (33.3%)
Current Clinical Role	
Physician	6 (40.0%)
Nurse Practitioner	1 (6.7%)
Psychologist	4 (26.7%)
Social Worker	3 (20.0%)
Therapist	1 (6.7%)

### Addressing the Individual Needs of Patients and Families

HCPs described how they tried to address the unique characteristics of each autistic adolescent and their family, resulting in an individualized approach to guidance on pursuing independent driving. When reflecting on engaging a family on the topic of driving, one HCP succinctly described what they thought it takes to drive: “intellectual capacity to pass the written test, the motor coordination to pass the driving test, or the executive function to be safe on the road.” (HCP #1). However, HCPs described how patient characteristics that could be protective and risk factors are part of the decision-making for families.

HCPs’ perceived strengths of autistic adolescents included how some autistic adolescents have strong visual-spatial skills and proclivity for memorizing directions, assets for independently navigating roadways. Adolescents’ hypervigilance and awareness of surroundings were perceived to result in attentiveness to the roadway with the ability to quickly react to hazards. HCPs noted a strong ability to memorize road rules while also being highly rule abiding to safety may help autistic youth set and achieve driving goals. It was acknowledged that patients with lower support needs may adapt to driving well and pursue independent licensure similarly to their non-autistic peers. Many autistic patients were perceived as highly motivated for independence, and therefore HCPs believed they should be prepared to support the youth and family to pursue driving and celebrate driving as an important developmental milestone.

I think in my experience working with young adults with autism, they very much more celebrate competency. Whereas somebody without a diagnosis it’s a ho-hum event to get a driver’s license or sometimes it may even be more of a chore or a burden. I think everything that’s achieved just gets – at the individual level, it’s celebrated a little bit in a healthier way – I should say. – HCP #2

Alternatively, some HCPs perceived characteristics in autistic adolescents that may make driving challenging. These include poor motor coordination; impulsivity and distractibility; poor social recognition/inability to read social cues or interpret ambiguous situations; poor reaction time; unpredictable behaviors (such as aggressive response); issues with executive functioning; and co-occurring conditions, particularly anxiety.

Well, I think anxiety is the biggest one and I think that feeling overwhelmed by the sheer possibilities. I’ve actually worked with a lot of kids on the spectrum who report that they kind of get frozen because they think, well, this could happen, but if I do this, that could happen. And so the thought process, I think, is overwhelming. And as we know, driving is a multitasker’s dream. And I think that’s probably one of the biggest factors. It is the idea of anxiety and feelings of overwhelmingness. – HCP #3

Less commonly perceived challenges discussed by HCPs included: poor sleep hygiene; seizures; and the need for different instructional styles due to mild intellectual disability. Some autistic traits were both potential strengths and weaknesses. For example, strict rule-following could lead to increased safety or make adapting to social cues on the road difficult.

HCPs indicated they have more difficult interactions in conversations regarding driving if the adolescent or family are not in agreement, or if the HCPs’ assessment and recommendations do not align with the caregiver’s or adolescent’s self-perceptions. These situations required HCPs to adapt their guidance and often to navigate difficult conversations with no preparation time. In some cases, HCP-family interactions were the first ones the parent and adolescent engaged in on driving. Some families were not prepared to talk about driving, with the indication that their adolescent is not ready, and they will return when they are ready to discuss driving.

…the mom has said, oh, we’ve been talking for many, many years about the possibility of driving in the future. And we’re not sure that we’re ready …. Or maybe it will in the future, but we have a lot to prepare for…. So that was the perfect contract of a family that did have resources, was aware of it, had prepared for it and the kid was completely fine with it because it was not – it didn’t catch her off guard. And she had enough insight to say, yeah, I’m not ready to drive, I’m not there yet, maybe someday. – HCP #4

### In-the-Moment Guidance and Referral Out

HCPs adapted their practices and guidance based on readiness and skills/abilities of patients and families. They described their role to be assessment of the patient’s individual characteristics noted previously, in addition to medical history and co-occurring conditions, and the skills needed to drive. Their assessment often led to “in-the-moment” of clinical encounter conversations and guidance, as well as referrals out to other experts.

HCPs indicated they had driving-related discussions with patients who were 15–23 years of age, but rarely with younger patients. One HCP described “having their routine,” in which they go through any required forms, talk with the adolescent and parents, consult with their therapist, and have prompts for the patients. Some perceived their role as helping the adolescent and parent navigate barriers and facilitate open communication. This sometimes translated into helping the family realize the nearing of a transition to adulthood.

Some HCPs described preparing in advance for specific patient encounters, whereas others spoke more about addressing issues in real-time during the visit as topics emerged. They explain the process of getting a permit and learning to drive with patients, while others talked through the different anxieties and concerns patients experienced, answering questions, and guiding families. Example education topics included the process for medical clearance for seizures, accommodations for the permit test, and the importance of medication adherence, if prescribed. Some discussed the legal contract of the learner’s permit and outlined ground rules for driving including no drinking and driving. HCPs also mentioned checking on the adolescent’s motivation to drive and using that to guide the direction of conversations.

Some HCPs implemented practices with families that included role playing, mindfulness, planning how to handle certain situations (e.g., stuck in traffic), adding driving to an Individualized Educational Plan (IEP), suggesting educational videos about driving (e.g., YouTube), and reviewing websites together. The HCPs roles in these interactions ranged, perhaps due to the nature of the type of HCP and the volume of autistic adolescents seen in their practice. HCPs felt they often played a role in supporting dialogue or conversation between adolescents and parents, especially during in-person office visits, to help families come to consensus about licensure and driving.

So it’s a kiddo who actually the family went ahead and decided that they were going to get him his permit, but now they’re facing a lot of difficulty as they try to get him to drive because he doesn’t actu﻿ally feel comfortable driving while the family does – like he’s ready. So it was a conversation around what are some of the reasons you’re uncomfortable, and a lot of them actually had to do with like, well, every day I don’t read social cues right, so what if I don’t read the ones on the road right, they might have really serious consequences and kind of just talking through things around that and how to make the decision of whether or not his discomfort is a valid concern or if it’s related to a different diagnosis of anxiety and eventually getting to the point of talking about some of this sounds like really reasonable concerns that all teenagers have about driving and not just you and really weaving it to be a family discussion that I can guide with some questions. – HCP #5

Many HCPs mentioned referring patients and families to other resources, including driving rehabilitation programs, driving instructors, and Office of Vocational Rehabilitation (OVR) transportation training. Sometimes this also meant pulling in additional clinicians, referring patients to their primary care provider, or pulling in other specialists who may have more resources.

I said, well, I’m gonna have to talk to his therapist and I am concerned about – he’s still getting some physical and occupational therapy at school and I’m concerned about his challenge and his capability to work the steering wheel, parking brake, and regular brake. I would like to consult with his physical therapist and, once again, I think we should be looking at a program that will work with him to make sure that he’s safe to be on the road. – HCP #6

Several HCPs mentioned referring to driving-related specialists, including driver’s education instructors and rehabilitative driving programs, which can evaluate readiness and build skills for licensure and safe driving.

### Resources: Present and Future

Many HCPs discussed current use and a “wish list” of resources to counsel and support patients and families. Current resources utilized ranged from reviewing available statistics related to driving and educational materials orienting the adolescent to driving, to gathering information from IEPs and ongoing services. Gathering information from psychologists, occupational therapists, rehabilitation/driving specialists, and schools informed HCPs about how they counsel families for driving and if they sign any required medical clearance forms. Some HCPs deferred to parents’ wishes for driving (or not-driving) and used resources to help guide the family in deciding collaboratively with their adolescent. HCPs described how some parents feel they have decided but look to the provider to help connect with resources. Some providers consult local programs to know what services can support their patients who are learning to drive.

There was a strong consensus among HCPs that no “gold standard” existed for resources to guide families of autistic adolescents on the path to licensure. However, multiple HCPs mentioned directing families to the Autism Speaks website and toolkits and mentioned using the CHOP patient family materials, including the health system’s general adolescent driver safety handouts. Some resources are “often hit or miss” as they sometimes do not apply to all autistic adolescents, or a limited set of tried-and-true resources like handouts were noted to be outdated. Some HCPs mentioned typically finding resources on their own via a Google search, exploring and networking, or hearing from patients what they themselves used. Finally, one HCP pointed out that anything helpful to the general population is also helpful to the autistic population.

Many HCPs expressed a “wish list” for more resources. This ranged from access to another practitioner they could turn to for support in conversations, such as a social worker or autism family coordinator, to a combination of handouts and websites tailored to the specific issue of autistic adolescents and independent driving. Multiple HCPs also expressed the need for a Driver’s Education program specifically for autistic individuals, with a dedicated and skilled instructor who understands autism, to which providers could confidently refer patients. Providers also felt it would be helpful to include a list of local instructors who are trained to teach autistic adolescents. Several HCPs mentioned that virtual driving simulation may help to assess readiness to drive, potentially by measuring reaction time, distractibility, and other skills while another mentioned simulation for practice/lessons. Other ideal or desired resources mentioned less frequently included videos, workshops, training on how to interact with law enforcement, role play opportunities, and parent support sessions.

HCPs also gave input on how resources should be shared. They emphasized that resources must be shared to specialties beyond those who typically see autistic patients and publicized throughout healthcare networks. Health system-specific suggestions included incorporating resources into electronic health records accessed by HCPs and families.

But I also think that it’s an example of how community resources don’t always support this important thing. This had just never come up and the family was not prepared to talk to the kid about this. And he didn’t have a psychiatrist or developmental pediatrician or a psychologist, he didn’t really have an autism provider, because he was high functioning, and doing well and didn’t really need it until he had a seizure. So there wasn’t anyone ushering the family through that. And it was looming, he was almost 16 years old and wanting to drive and no one had ever dealt with this. And so I think building up the community supports and the community resources and putting it on the radar of teachers and therapists and social workers. – HCP #4

## Discussion

In this qualitative study using semi-structured interviews with 15 HCPs, it was suggested that planning for the transition to independent driving requires an individualized approach for autistic adolescents and their families. Not every adolescent will be ready to get a license, but HCPs who discuss the learning to drive and independent licensure process with families want to provide guidance in real-time that fits the developmental trajectory of the adolescent. Although there are times when the HCPs have the tools to guide families, referral out and other resources may be needed. These HCPs described a need for specific resources for the autistic population given the unique strengths and challenges. Although general adolescent driving resources can be a starting point, there is the opportunity to tailor, adapt, and extend what is done with non-autistic adolescents to be more appropriate for autistic adolescents pursuing independent licensure.

HCPs identified the importance of knowing and assessing strengths and challenges autistic adolescents may experience on the path to independent driving. Such an individualized approach is like what specialized driving instructors noted when describing behind-the-wheel instruction to autistic adolescents (Myers et al., 2021). HCPs should be prepared to work with autistic patients and families as they typically have long-standing relationships that can support success in navigating developmental milestones (Cooley et al., 2011). They are apt to assess strengths their autistic patient may bring to the learning-to-drive process and potential areas of challenge that may require additional support and training. Continued efforts to develop decision support tools and other clinical pathways may help HCPs feel better prepared for collecting and making recommendations based on this information. For example, adolescents that have co-occurring conditions that may need additional support and time in the learning to drive process (Almberg et al., 2015). HCPs should be ready to help families use that information as they figure out how to engage in independent driving.

The description of the counseling during the clinical encounter coupled with referring out connects to the individualized approach of knowing what an adolescent and their family need during this developmental transition. Importantly, HCPs often found themselves initiating conversations with adolescents and their families about driving, which could be challenging to navigate due to the lack of accessible resources and incomplete clinical information. HCPs need awareness of who they can connect patients to for resources or additional assessments from other providers. For example, for primary care providers, there is already little time in a clinical encounter for autistic adolescents and families with their HCP to add an additional assessment burden. Instead, HCPs should have the resources to identify which other specialists may conduct such assessments and relay information to help coordinate care. This could include occupational therapists, certified driving rehabilitation specialists, psychologists, or social workers. A recent study reported that only 8% of HCPs feel comfortable taking on the burden of assessment, but yet only 25% of HCPs refer their patients to outside resources, highlighting an opportunity to intervene and close this gap (Sartin et al., 2021). There may be one HCP who has the longest standing relationship with the adolescent and family who may be poised as the professional who can take in the information and then synthesize it to provide guidance to the family. This approach can blend what is important on individualization with ‘in-the-moment/referral out.’

HCPs in these interviews clearly described missing, but needed, resources for their autistic patients and families around the learning to drive process. There is precedent for HCPs to have a role in this transitional milestone (Mirman et al., 2018). Past interventions, however, were not developed with autistic adolescents as the target audience. Though there are programs being developed (Baker-Ericzén et al., 2021), given the specific challenges autistic adolescents face, there is a need for autism-specific guidance and resources. Collaboration on resource development with autistic adolescents and young adults and their families can help support implementation, such as the acceptability, adoption, and appropriateness of such resources (Proctor et al., 2011). Based on these interviews, resources could include a tailored menu to help the family find what they need and include the step-by-step process of learning to drive and getting a license. It could also integrate visual and written instructions along with suggested practice methods, what skills are necessary, and guidelines for parents, such as what to expect as an adolescent starts driving and potential conversation topics.

### Limitations

This study has several limitations. First, we interviewed HCPs from a singular healthcare system and Centers designated as Autism Centers for Excellence within Philadelphia County. These HCPs served patients in Philadelphia and surrounding counties, and thus there may be perspectives of HCPs that serve other geographic areas that may be different and not elicited in these interviews. The perspectives of the HCPs were reflective and retrospective of patients that had often transitioned to adult care and HCPs were unaware of the licensure and driving outcomes. Although the HCPs had a variety of clinical roles, singular representation from disciplines occurred and therefore saturation may not have been reached within a given clinical role. However, thematic saturation across interviews was reached.

## Conclusions

From these qualitative interviews with 15 HCPs, current strategies and perceived needs for providing guidance about independent driving to autistic adolescents and families were identified. HCPs use an individualized approach for guidance in the transition to independent driving, considering the unique needs of their autistic adolescent patients and families. These HCPs identified a clear need for and recommendations for additional resources and guidance they can use in support of independent driving when appropriate for their patients and families. Further work is needed to develop and deliver driving-related resources for HCPs so that they can optimally support autistic adolescents and their families as they are considering whether to pursue independent driving.

## Abbreviations

HCP: Healthcare providers
